# Rapid maxillary expansion in patient with obstructive sleep apnea: case report

**DOI:** 10.5935/1984-0063.20220026

**Published:** 2022

**Authors:** Rita Catia Bariani, Fauze Ramez Badreddine, Lucia Hatsue Yamamoto, Flavio Toshiki Shido, Daniela Pimentel Machado Renofio Hoppe, Sergio Tufik, Gustavo A. Moreira, Mario Cappellette, Reginaldo Raimundo Fujita

**Affiliations:** 1 Universidade Federal de São Paulo, Department of Otorhinolaryngology and Head and Neck Surgery - São Paulo - SP - Brazil.; 2 Universidade Federal de São Paulo, Department of Psychobiology - São Paulo - SP - Brazil.

**Keywords:** Sleep Apnea, Obstructive, Palatal Expansion Technique, Sleep Apnea Syndromes, Craniofacial Abnormalities, Snoring. Child

## Abstract

The aim of this case report was to evaluate the polysomnography indices, air space in the oropharyngeal region and quality of life scores using the OSA-18 questionnaire in a patient diagnosed with obstructive sleep apnea before and after rapid maxillary expansion (RME). It is a case report with a male patient, seven years old, with maxillary hypoplasia, who underwent adenotonsillectomy surgery two years ago, had restless sleep, snore more than five times a week. Pre- and post-treatment diagnostic tests were performed, including nasofibroscopy, polysomnography, computed tomography, orthodontic records and the OSA-18 quality of life questionnaire. The treatment consisted of RME with Hyrax maxillary expander. After six months, the exams were redone. The polysomnographic record before treatment: IAH 2.8/h, after treatment 0.5/h. We concluded that rapid maxillary expansion (RME) in children with OSA appears to be an effective treatment.

## INTRODUCTION

The term sleep-disordered breathing includes a variety of pathologic conditions ranging from primary snoring and upper airway resistance to obstructive sleep apnea. Obstructive sleep apnea (OSA) in childhood is characterized by intermittent partial or complete collapse of the upper airway (obstructive hypopnea or apnea).^1^ Childhood OSA is common and has a multifactorial origin. In children free of syndromes or comorbidities, its main etiology is adenotonsillar hypertrophy. In addition to large tonsils and adenoids, children with OSA may present narrow upper airway as a result of having narrow long faces, maxillary constriction, high arched palate or some degree of mandibular retrusion^2^. The diagnosis and planning of the appropriate treatment for children with OSA may require the involvement of various medical specialties, depending on the complexity, severity, and persistence of OSA^2^. In the absence of treatment, OSA can affect neurocognitive abilities, school performance, behavior, and the cardiovascular system - having a potentially significant impact on the child’s quality of life. Therefore, early diagnosis and treatment of OSA is extremely important and can help to avoid possible complications^3^.

Most healthy children respond favorably to adenotonsillectomy as a first-line treatment, but 20% of children have OSA refractory to adenotonsillectomy (AT)^4^. Limited studies have suggested that variables such as obesity, asthma, impaired facial skeletal growth or severe craniofacial anomalies, a high apnea hypopnea index (AHI) and male gender can be risk factors for persistent OSA after AT.^5,6^ Additional treatment includes corticosteroids^7^, (RME)^8^, myofunctional therapy^9^, behavioral measures related to diet and weight loss^10^, continuous positive airway pressure^11^, or combined interventions^4^

RME is a dentofacial orthopedic treatment procedure commonly adopted in young patients for the treatment of constricted maxillary arches.^8^ Several studies have shown the short-term efficacy of orthodontic treatment with a rapid maxillary expander, and evidence of a significant improvement of OSA^12^. The objective of this case report was to demonstrate the effectiveness of RME as a form of treatment for OSA refractory to adenotonsillectomy.

## CASE REPORT

### Diagnosis and etiology

An otolaryngologist referred a 7-year-old Caucasian male patient in good general health for orthodontic treatment. The patient had already undergone adenotonsillectomy three years previously but had restless sleep and snored more than five times a week. A nasofibroscopy exam confirmed that there was no pharyngeal or palatal obstruction. Before and after treatment, an orthodontic evaluation, polysomnography, and computed tomography were performed. In addition, the OSA-18 questionnaire was completed, and all orthodontic documentation was examined.

### Orthodontic analysis:

**Figure 1.**
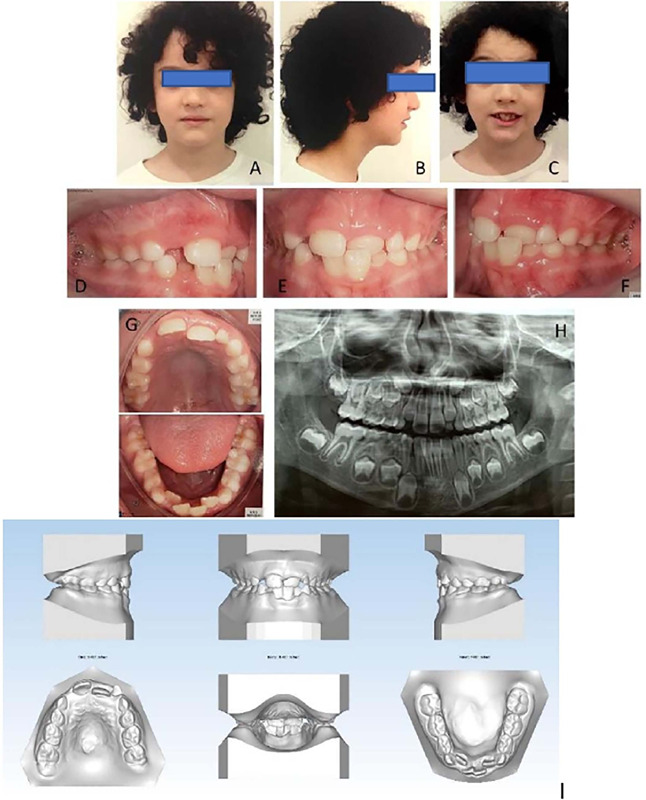
Pretreatment. (A-B-C) extraoral photographs; (D-E-F-G) intraoral photographs; (H) orthopantomograph (I) intraoral models.

In frontal and lateral facial analyses, a small facial asymmetry was observed with no lip seal, a practically nonexistent nasolabial groove, a straight profile, and a normal chin and neck line (Fig. 1 A, B, C). The occlusal evaluation showed that the patient was in the mixed dentition phase presenting Angle Class I malocclusion, anterior crossbite, maxillary atresia, lack of space for the upper canines, and lower midline deviation to the left (Fig. 1 D, E, F, G, I).

Panoramic radiography (Fig. 1 H) showed that all teeth were healthy, with good bone morphology, but no space for the upper canines. Cephalometric analysis indicated a normodivergent facial pattern, increased lower facial height, retrusive lingually tipped upper incisors and protruding lower incisors, although the incisors were well positioned in their bony bases.

### Tomography evaluation

Computed tomography (CT) scans were performed at the institution’s Diagnostic Imaging Department, using a multislice device (Philips® Brilliance CT 64-channel device). CT was performed pre- (T1) and post-RME (T2), with a period of approximately 6 months between then. The images were used to assess the impact of RME on the oropharynx with measurements of its volume in cubic millimeters. The images were analyzed on the Dolphin Imaging 11.5 Premium Software using the Airway Volume tool. It was delimited by the following cephalometric points: posterior nasal spine, Basion (Ba), second cervical vertebra (C2), and hyoid bone.

### Polysomnography

We used the definitions of American Academy of Otolaryngology Head-Neck Surgery Foundation (AAO-HNSF)^13^ as follows: Obstructive sleep-disordered breathing (oSDB) is a clinical diagnosis that includes a clinical spectrum ranging from snoring to obstructive sleep apnea. (OSA) = oSDB with an abnormal polysomnography with an obstructive apnea/hypopnea index (oAHI) > 1 event/hour. This definition implies that a diagnosis of OSA cannot be established without objective testing PSG.^14^

### OSA-18 Questionnaire

OSA-18 is a questionnaire designed to assess quality of life in children with apnea and has been validated in Portuguese^15^. OSA-18 consists of 18 questions divided into five domains: sleep disorder, physical symptoms, emotional symptoms, daytime function, and caregiver concerns. Each item has a score of 7 points (1 – “never” to 7 – “always”)^15^. A score of less than 60 indicates a mild impact on quality of life, 60-80 a moderate impact, and above 80 a serious impact.^15^

### Intervention

**Figure 2.**
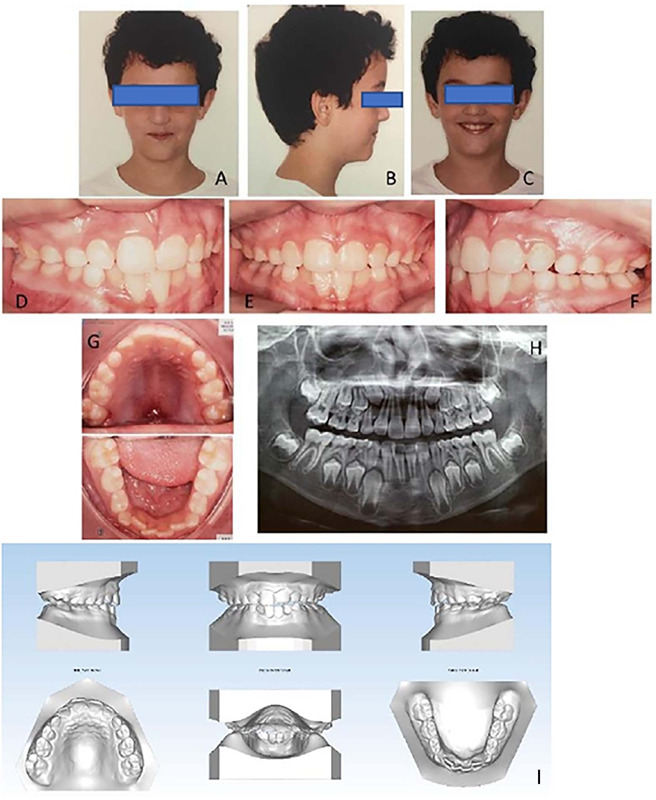
Post-treatment. (A,B,C) extraoral photographs; (D,E,F,G) intraoral photographs; (H) orthopantomograph; (I) intraoral models.

After the exams, the patient underwent treatment with a Hyrax device for RME, an orthodontic-orthopedic procedure that uses fixed appliances anchored in the posterior teeth and welded to an expander screw located in the palate region. The activation protocol of the appliance used was as follows: after placement, the patient remained with the device for one week without activation to get used to it. The device was then activated by an orthodontist with 6/4 turns of the expander screw. The child’s parents were then instructed on how to operate the device and told to make two activations (2/4) per day, with no interval between them until complete correction of the transverse problem. During the active phase of RME, the patient returned weekly to assess the amount of maxillary expansion and check the correct activation of the screw by the parent and record any clinical signs, such as the diastema between the upper incisors. The procedure was continued until contact was observed between the inclined planes of the buccal slopes of the upper first molars and the lingual inclined planes of the buccal cusps of the lower first molars, and there was transverse compatibility of the maxillary bone base with the Wala edge of the mandibular bone base. The patient continued with the Hyrax expander passively for six months after the last activation. This is the retention period for new bone formation in the midpalatal suture. After this period, the maxillary disjunction device was removed. After removing the device, the parents completed the OSA-18 questionnaire, and the child again underwent orthodontic exams, polysomnography, and computed tomography (Fig. 2).

## RESULTS

**Figure 3.**
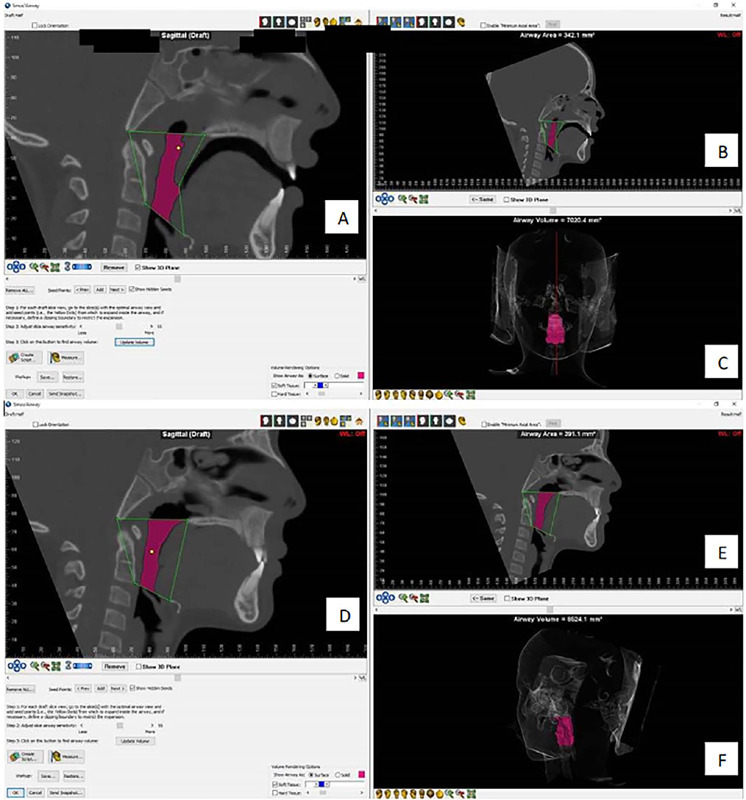
Volume of the oropharynx. (A,B,C) pretreatment; (D,E,F) post-treatment.

Treatment with RME increased transverse maxillary expansion and reduced maxillary constriction. In addition to the benefits of balanced occlusion, this increased the nasopharyngeal airway and reduced nasal resistance, facilitating nasal breathing. Computed tomography demonstrated an increase in the oropharyngeal air space from 821mm³ before treatment to 862mm³ after treatment (Fig. 3). Before treatment, the overall average score for OSA-18 was 125 (severe impact); The highest mean scores in the OSA-18 were in the domains “sleep disorders”, followed by “caregiver concerns” and “physical symptoms”, domains which involve questions about the most common aspects of apnea, such as snoring, asphyxia, restless sleep, mouth breathing, upper airway infection, difficulty feeding, and parental concern about their child’s health. The results of the OSA-18 questionnaire after RME showed improved scores in all five domains with a score of 45, showing improvement in quality of life.

Polysomnographic data before and after RME are shown in Table 1. The obstructive apnea-hypopnea index improved from 2.8 to 0.5/h. All results confirmed the improvement in the patient’s clinical picture.

**Table 1.** Polysomnography characteristics.

Variables	Pre-treatment	Post-treatment
AHI	3.4 (nº/h)	1.1 (nº/h)
Obstructive AHI	2.8/h	0.5/h
Central AHI	0.6/h	0.6/h
AHI supine	1.9 (nº/h)	0.7 (nº/h)
AHI non supine	4.2 (nº/h)	1.8 (nº/h)
AHI maximum	25.6	13.7
AHI minimum	13.5	11.1
RDI	3.4 (nº/h)	1.1 (nº/h)
Baseline SpO2	95.3%	94.3%
Mean SpO2	93.8 %	94.0 %
Minimum SpO2	87.0%	89.0%

AHI: apnea/hypopnea index; RDI: respiratory disturbance index; SpO2: oxygen saturation.

## DISCUSSION

Effective treatment for OSA in children should be focused on one or more risk factors to help cure the obstruction.^16^ RME and adenotonsillectomy are proven treatments of (OSA) in children.^17^Adenotonsillectomy is not always successful in controlling (OSA) in children due to variables such as obesity, asthma, impaired facial skeletal growth, craniofacial anomalies, and a severe apnea hypopnea index (AHI).^5,6^ Orthodontic treatment may be a helpful adjunct and be a particularly important treatment for children with impaired facial skeletal growth or craniofacial anomalies.^4^

Treatment with RME in this patient corrected the skeletal discrepancy and increased airway volume, thereby reducing the apnea hypopnea index (AHI) and the associated complications and improved the quality of life. In agreement with our findings, a number of studies have concluded that treatment with RME for a period of 4 to 6 months can improve the AHI index.^18,19^

One study looked at the effects of RME versus AT: non-obese children presenting with narrow jaws and both adenoid and tonsillar enlargement were assigned randomly to surgical or orthodontic treatment. With the exception of one child who improved with orthodontic treatment alone, all subjects required both treatments to see a significant improvement of OSA.^17^ Villa et al ^20^ The authors selected 52 children with OSA and divided them into 3 groups. Children with milder disorder were referred to RME, while those with more severe disorder were referred for adenotonsillectomy. After the interventions, there was reduction in the AHI in both procedures, yet children submitted to surgery and still presenting apnea were submitted to RME, thus establishing the third treatment group. There was improvement in the clinical picture of OSA in the three treated groups. According to the authors, age at onset and severity of OSA should be the main factors involved in treatment selection. In this study, the patient with OSA had already been treated with adenotonsillectomy two years earlier but still had a low quality of life and behavioral problems such as mood changes, hyperactivity and disciplinary problems. This is important, because non-diagnosed primary snoring and OSA may cause significant consequences for health. Studies to evaluate treatment alternatives for children with refractory sleep-disordered breathing (SDB) after late AT are scarce.

This case report may aid, cautioning professionals to provide primary attention to the risk factors and diagnosis of snoring and OSA caused by late adenotonsillectomy.

The early treatment of craniofacial abnormalities can prevent the development of long-term respiratory failure, impacting the quality of life in adulthood.^4^

## CONCLUSION

RME should be considered a treatment option in children with OSA and maxillary hypoplasia. Parents, patient and interdisciplinary cooperation in care are important in this type of treatment.
